# Human pluripotent stem cell‐derived epicardial progenitors can differentiate to endocardial‐like endothelial cells

**DOI:** 10.1002/btm2.10062

**Published:** 2017-05-22

**Authors:** Xiaoping Bao, Vijesh J. Bhute, Tianxiao Han, Tongcheng Qian, Xiaojun Lian, Sean P. Palecek

**Affiliations:** ^1^ Dept. of Chemical & Biological Engineering University of Wisconsin Madison WI 53706; ^2^ Dept. of Biomedical Engineering Biology and Huck Institutes of the Life Sciences, The Pennsylvania State University University Park PA 16802

**Keywords:** cardiac differentiation, endocardium, endothelial cells, epicardium, genome editing, human pluripotent stem cells

## Abstract

During heart development, epicardial progenitors contribute various cardiac lineages including smooth muscle cells, cardiac fibroblasts, and endothelial cells. However, their specific contribution to the human endothelium has not yet been resolved, at least in part due to the inability to expand and maintain human primary or pluripotent stem cell (hPSC)‐derived epicardial cells. Here we first generated CDH5‐2A‐eGFP knock‐in hPSC lines and differentiated them into self‐renewing WT1+ epicardial cells, which gave rise to endothelial cells upon VEGF treatment *in vitro*. In addition, we found that the percentage of endothelial cells correlated with WT1 expression in a WT1‐2A‐eGFP reporter line. The resulting endothelial cells displayed many endocardium‐like endothelial cell properties, including high expression levels of endocardial‐specific markers, nutrient transporters and well‐organized tight junctions. These findings suggest that human epicardial progenitors may have the capacity to form endocardial endothelium during development and have implications for heart regeneration and cardiac tissue engineering.

## 1 INTRODUCTION

The epicardium is the outermost mesothelium layer of the heart that is essential for both heart development and cardiac remodeling.^1^ During cardiogenesis, a subset of epicardial cells invades the underlying myocardium and contributes to various cardiac lineages, including cardiac fibroblasts and smooth muscle cells.^2^ A recent report also suggests an epicardial origin for a subset of cardiomyocytes,^3^ although the *in vivo* fate mapping studies used to draw this conclusion are susceptible to artifacts. More recently, human sinoatrial node cardiomyocytes have been derived from TBX18+ progenitor cells that also contribute to epicardial cells,^4^ but it remains unknown whether WT1+/TBX18+ epicardial cells can also contribute to cardiomyocyte populations. Similarly, the epicardial contribution to the developing cardiac endothelium remains controversial.

*In vivo* lineage tracing studies have shown that a subpopulation of coronary endothelial cells arise from the epicardium in the chicken,^5^ while studies in mice failed to identify a significant epicardial contribution to endothelial cells via fate mapping using the well‐known epicardial cell markers TBX18 and WT1.^3^, ^6^ Recently, Scleraxis (Scx) and Semaphorin 3D (Sema3D) were identified as markers of epicardial cells that contribute to both coronary vascular endothelium and cardiac endocardium.^7^ Zhang et al.^8^ identified natriuretic peptide receptor 3 (NPR3) as a specific endocardial marker and demonstrated their contribution of NPR3‐expressing endocardial cells to coronary vessels. The expression of WT1 in developing human fetal hearts follows a pattern starting at the epicardium and extending toward the lumen of the heart, and WT1 expression in endocardial cells nearly disappeared at week 20, suggesting WT1+ epicardial cells as a potential cell origin of endocardial endothelial cells.^9^ However, understanding of the developmental progression of human epicardial cells to endothelium and endocardium is still extremely limited, mainly due to ethical and logistical challenges of tracing cells in the developing human heart and the lack of an *in vitro* human model to study the epicardial‐to‐endothelial transition.

Over the past 3 years, multiple labs have developed robust protocols to generate epicardial‐like cells from human pluripotent stem cells (hPSCs) by manipulating Wnt, bone morphogenetic protein and retinoic acid signaling pathways that are important for *in vivo* epicardium development.^10^, ^11^, ^12^, ^13^ While hPSC‐derived epicardial cells from different protocols have the potential to differentiate into smooth muscle cells and cardiac fibroblasts both *in vitro* and *in vivo*, none of them have yet been shown to form endothelial cells so far. To develop an hPSC model to study epicardial cell differentiation to endothelial cells, we first generated CDH5‐2A‐eGFP knock‐in hPSC lines and differentiated them into self‐renewing WT1+ epicardial cells which gave rise to endothelial cells upon VEGF treatment *in vitro*. We also showed that the purity of epicardial‐derived endothelial cells is proportional to WT1 expression in a WT1‐2A‐eGFP reporter line. In addition, the resulting epicardial‐derived endothelial cells displayed many endocardium‐like endothelial cell (EEC) properties, including high expression levels of specific endocardial markers, nutrient transporters and well‐organized tight junctions. These findings demonstrate that hPSC‐derived epicardial cells have the capacity to differentiate to endocardial‐like cells for potential applications in heart tissue engineering and suggest that human epicardial cells may contribute to endocardial cells during cardiac development.

## 2 RESULTS

### 2.1 Generation of CDH5‐2A‐eGFP knock‐in reporter hPSCs via CRISPR/Cas9 genome editing

**Figure 1:**
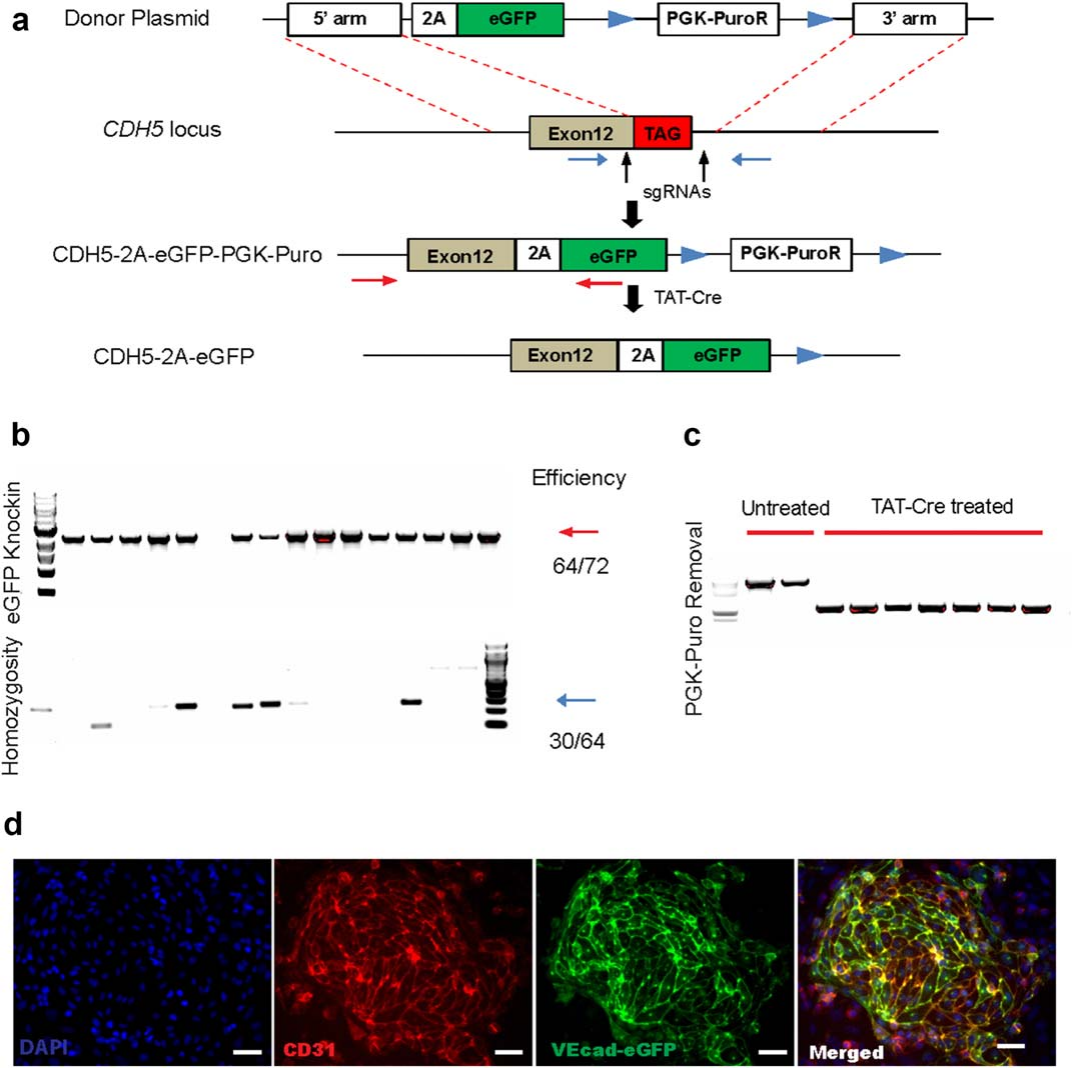
Generation of CDH5‐2A‐eGFP knock‐in H9 hESC lines using Cas9 nuclease. (a) Schematic diagram of the targeting strategy at the stop codon of the *CDH5* locus. Vertical arrows indicate sgRNA1 and sgRNA2 targeting sites. Red and blue horizontal arrows indicate PCR genotyping primers for assaying *CDH5* locus targeting and homozygosity, respectively. (b) Representative PCR genotyping of hESC clones after puromycin selection. The expected PCR product for correctly targeted *CDH5* locus is ∼3 kb (red arrows) with an efficiency of 64/72 clones. Correctly targeted clones underwent a further homozygosity assay. Clones with the PCR products of ∼200 bp are heterozygous (blue arrow), and those clones without PCR products are homozygous. (c) PCR genotyping of hESC clones after TAT‐Cre mediated excision of the PGK‐Puro cassette. Clones with PCR products of ∼1 kb are PGK‐Puro free, and those with ∼3 kb contain PGK‐Puro. (d) Representative CD31 and eGFP dual immunostaining images of CDH5‐2A‐eGFP hPSC‐derived endothelial cells after excision of the PGK‐Puro cassette. Scale bars, 50 µm

In order to better monitor the transition of hPSC‐derived epicardial cells to endothelial cells, we engineered the H9 human embryonic stem cell (hESC) line via CRISPR/Cas9‐catalyzed homology‐directed repair (HDR) and generated a homozygous CDH5‐2A‐eGFP knock‐in reporter cell line (Figure 1a). Two 2‐kilobase homologous arm sequences located before and after the *CDH5* stop codon were inserted into the Oct4‐2A‐eGFP donor plasmid^14^ and replaced the *Oct4* homologous arms. We then introduced the 2A‐eGFP sequence into the targeting sites by transfecting hPSCs with the CDH5‐2A‐eGFP donor plasmid and the Cas9/sgRNA plasmids. After puromycin selection, PCR genotyping showed that ∼90% (64/72) of the clones were targeted in at least one and ∼40% (32/72) in both alleles (Figure 1b). The homozygous clones were then subjected to TAT‐Cre recombinase treatment and the PGK‐Puro cassette was excised from CDH5‐2A‐eGFP (Figure 1c). CDH5‐2A‐eGFP‐targeted hPSCs after Cre‐mediated excision of the PGK‐Puro cassette were subjected to endothelial cell differentiation via a previous published protocol.^15^ Dual immunostaining with anti‐CD31 and anti‐GFP antibodies showed expression of eGFP in CD31+ cells (Figure 1d), demonstrating success in generating a *CDH5* reporter cell line for potential cell tracking or purification. We also successfully knocked the 2A‐eGFP cassette into the H13 hESC line (Supporting Information Figure S1).

### 2.2 VEGF signaling permits endothelial transition from hPSC‐derived epicardial cells

**Figure 2:**
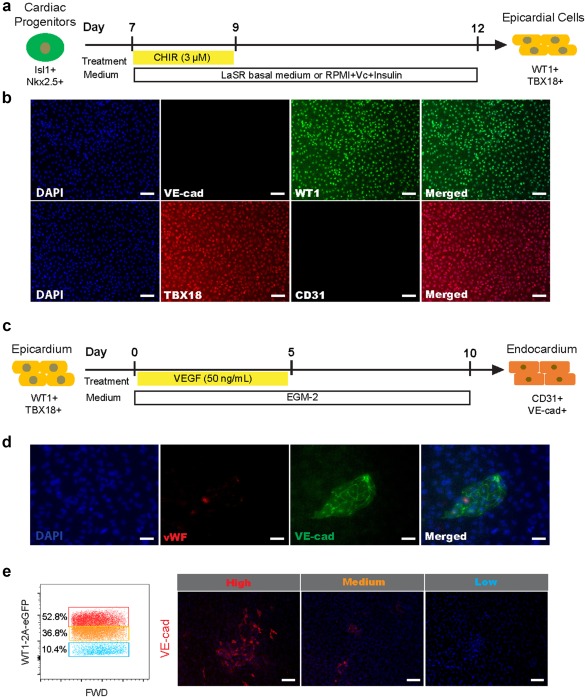
VEGF exposure directs a subset of hPSC‐derived epicardial cells to endothelial cells. (a) Schematic of the protocol for differentiation of hPSC‐derived cardiac progenitors to epicardial cells via CHIR treatment. At day 12, cells were subjected to immunostaining analysis of VE‐cadherin, CD31, WT1, and TBX18, and representative images are shown in (b). Scale bars, 100 µm. (c) Schematic of the protocol for differentiation of hPSC‐derived epicardial cells to endothelial cells via 5‐day VEGF treatment in EGM‐2 medium. After 10 days, cells were analyzed for vWF and VE‐cadherin expression by immunostaining (d). Scale bars, 50 µm. (e) WT1‐2A‐eGFP+ epicardial cells were FACS sorted according to the GFP signal (red: high (52.8 ± 3.3%), yellow: medium (36.8 ± 3.0%), and blue: negative (10.4 ± 0.7%)) and differentiated into endothelial cells as shown in (C). On day 10, differentiation cultures were subjected to VE‐cadherin immunostaining and representative images are shown. Scale bars, 100 μm

We previously demonstrated that temporal modulation of canonical Wnt signaling was sufficient to generate self‐renewing WT1 + TBX18+ epicardial cells from hPSCs.^10^ Treatment of undifferentiated hPSCs with the GSK3β inhibitor CHIR99021 resulted in mesoderm formation and subsequent inhibition of Wnt signaling via a Porcupine inhibitor directed the cells to ISL1 + NKX2.5+ cardiac progenitors. Treating the cardiac progenitors with CHIR99021 from days 7 to 9 of differentiation generated a virtually pure population of epicardial cells which did not express the endothelial cell markers CD31 and VE‐cad (Figure 2a,b). Upon 50 ng/ml VEGF treatment in EGM‐2 medium for 5 days, a small subset of these WT1 + TBX18+ expressing epicardial cells became VE‐Cad + vWF+ endothelial cells (Figure 2c,d). Next, we tested different concentrations of VEGF in generating endothelial cells from epicardial cells, and found that 50 and 100 ng/ml VEGF significantly improved the epicardial‐to‐endothelial transition compared to the no‐VEGF control (Supporting Information Figure S2a,b). To further confirm these endothelial cells arose from WT1+ epicardial cells, we generated ES03 WT1‐2A‐eGFP knock‐in hPSCs and differentiated them into WT1+ epicardial cells,^10^ which were expanded for several passages in LaSR basal medium containing 0.5 µM A83‐01, which permits expansion and inhibits dedifferentiation of hPSC‐derived epicardial cells,^10^ and then sorted the cells into populations expressing high (52.8 ± 3.3%), medium (36.8 ± 3.0%), and undetectable levels (10.4 ± 0.7%) of WT1 by fluorescence activated cell sorting (FACS) (Figure 2e). These populations were then subjected to 100 ng/ml VEGF in EGM‐2 medium. Interestingly, we observed the most VE‐cad+ cells from epicardial cells exhibiting high expression of WT1 while the WT1‐cells did not generate cells expressing detectable VE‐cad (Figure 2e, Supporting Information Figure S2c). This is consistent with previous avian studies demonstrating epicardial cells give rise to endothelial cells.^16^, ^17^, ^18^

### 2.3 Endothelial cells from WT1+ cells display endocardial properties

**Figure 3:**
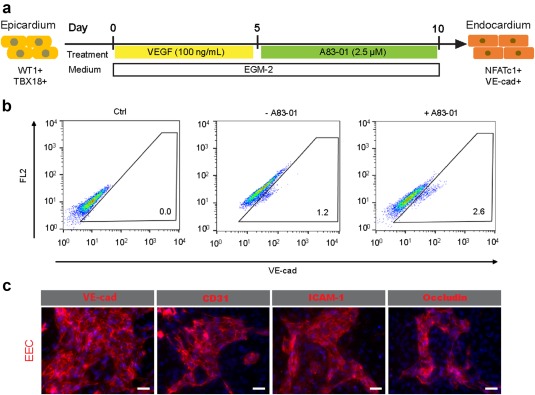
WT1+ cell‐derived endothelial cells display endocardial‐like properties. (a) Schematic of the process for differentiation of hPSC‐derived epicardial cells to EECs. (b) On day 10, cultures differentiated in the absence and presence of A83‐01 were subjected to flow cytometry analysis of VE‐cadherin and representative dot plots are shown. Numbers represent the percentage of cells in the indicated gated regions. (c) Representative immunostaining images of endothelial markers VE‐cadherin, CD31, and ICAM‐1, as well as tight junction protein occludin in cells differentiated as shown in (a). Scale bars, 50 µm

**Figure 4:**
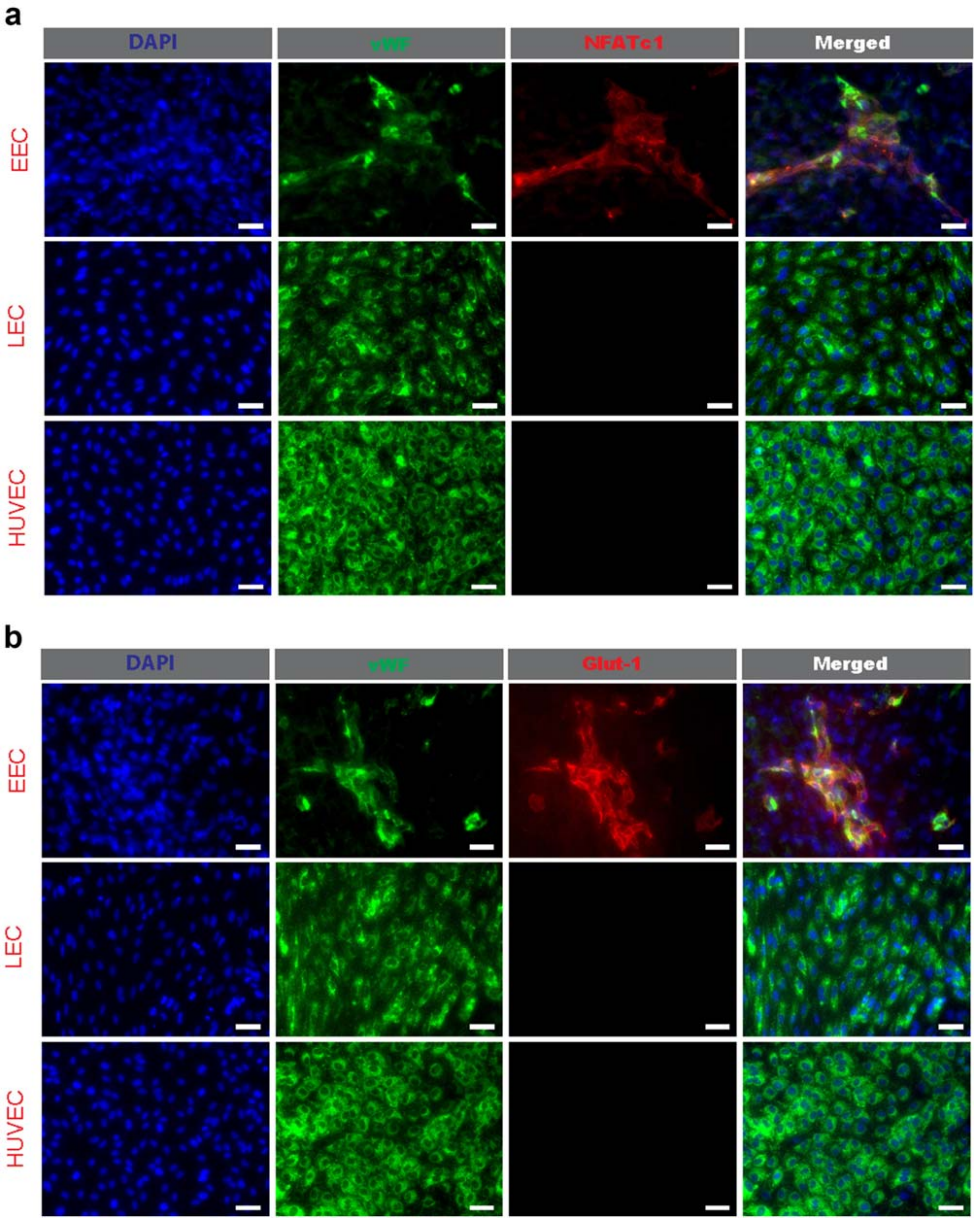
Immunostaining comparison of endocardial cell marker NFATC1 (a) and nutrient transporter protein Glut‐1 (b) in different types of endothelial cells. EEC: hPSC‐derived endocardial endothelial cells; LEC: Endothelial cells differentiated from hPSCs in LaSR basal medium; HUVEC: Human umbilical vein endothelial cells. DAPI and vWF co‐staining indicate nuclei and endothelial cells, respectively. Scale bars, 50 µm

Previously, TGFβ inhibition via A83‐01 treatment was shown to promote proliferation of hPSC‐derived endothelial cells.^19^ To increase the yield of endothelial cells from hPSC‐derived epicardial cells, we treated the cells with 2.5 µM A83‐01 for 5 days after VEGF treatment. A83‐01 treatment doubled the purity of VE‐cad+ cells to 2.6%, as expected (Figure 3a,b). The resulting epicardial‐derived endothelial cells expressed hallmark endothelial cell markers CD31, ICAM‐1, and vWF, as well as the tight junction protein occludin (Figure 3c, Supporting Information Figure S3). Intriguingly, these epicardial‐derived endothelial cells strongly expressed the EEC‐marker NFATc1^20^, ^21^, ^22^ and the nutrient transporter protein Glut‐1, compared to endothelial cells differentiated from hPSCs through a nonepicardial pathway in LaSR basal medium (LEC)^15^, ^23^ and human umbilical vein endothelial cells (HUVEC) (Figure 4a,b). In summary, A83‐01 treatment increased the purity of endocardial‐like endothelial cells from WT1+ cells, and they displayed specific EEC markers. This is consistent with an *in vivo* report that epicardial cells can give rise to NFATc1+ endocardial cells during mouse heart development.^7^

### 2.4 Characterization of hPSC‐derived EECs

**Figure 5:**
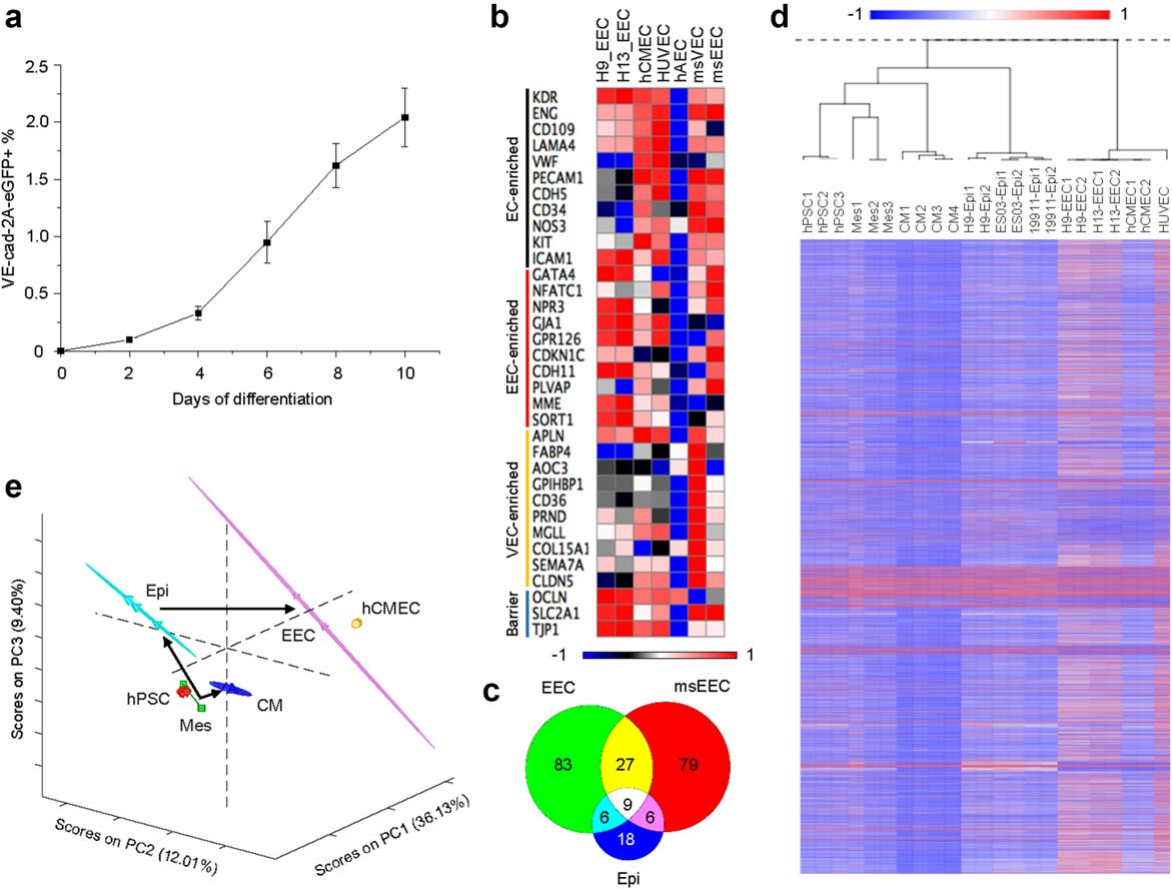
Molecular profiling of hPSC‐derived EECs. (a) H13 VE‐cad‐2A‐eGFP‐derived epicardial cells were differentiated into EECs according to Figure 3a, and cells were subjected to flow cytometry analysis for GFP expression at the indicated days. Data represent mean ± SEM of five independent replicates. (b) Expression of the endothelial subtype marker genes quantified by the normalized RPKM from RNA‐seq. Markers of different subtypes are indicated with colored bars. Black: standard endothelial markers; red: EEC‐enriched markers; yellow: cardiac vascular endothelial cell (VEC)‐enriched markers; blue: barrier‐related genes. hCMEC: human cardiac microvascular endothelial cells; HUVEC: human umbilical vein endothelial cells; hAEC: human aortic endothelial cells; msVEC: mouse cardiac vascular endothelial cells; msEEC: mouse endocardial endothelial cells. (c) Venn diagram showing the number of pathways which were enriched in different cell types relative to undifferentiated hPSCs. (d) Hierarchical clustering analysis of RNA‐seq expression data of hPSCs, hPSC‐derived mesoderm (Mes), cardiomyocytes (CMs), epicardial cells (Epi), and EEC, as well as primary hCMEC and HUVEC. (e) 3D scores plot of first 3 principal components (PCs) from PC analysis (PCA) of RNA‐seq data. The ellipses show the 95% confidence limits and each data point corresponds to a unique biological sample. Black arrows show the differentiation progression from mesoderm to CMs and epicardial cells, from which EEC arise

In order to further confirm the endocardial identity of these putative hPSC‐derived EECs, we differentiated VE‐cad‐2A‐eGFP knock‐in cell lines into epicardial cells and then subjected these cells to sequential VEGF and A83‐01 treatment as shown in Figure 3a. On day 10, both H9 and H13 cell lines yielded ∼ 2.5% VE‐cad+ cells (Figure 5a, Supporting Information Figure S4), which were then purified via FACS for VE‐cad expression and subjected to RNA‐seq analysis. We first compared the expression of known endothelial lineage‐specific signature genes in hPSC‐derived EECs, human cardiac microvascular endothelial cells (hCMECs), HUVECs (GSE54384), human aortic ECs (hAECs), mouse cardiac vascular endothelial cells (msVECs),^8^ and mouse EECs (msEECs)^8^ (Figure 5b). EEC marker genes such as GATA4,^24^ NFATc1,^20^, ^21^, ^22^ NPR3^8^ and GPR126,^8^ and tight junction genes and nutrient transporters including occludin (OCLN),^25^ ZO1 (TJP1),^25^ and GLUT‐1 (SLC2A1),^25^ were highly enriched in hPSC‐derived EECs and msEECs, while cardiac vascular endothelial cell marker genes including APLN,^8^ FABP4,^8^ and GPIHBP1^8^ were highly enriched in hCMECs and msVECs. We also performed gene set enrichment analysis (GSEA) to identify significantly enriched pathways (*p* < .05) in each cell type relative to undifferentiated hPSCs. We compared the differences and similarities in the enriched pathways among hPSC‐derived EECs, hPSC‐derived epicardial cells, and mouse EECs (Figure 5c). We observed that while 9 pathways were commonly enriched in all three cell types, hPSC‐derived EECs shared 27 enriched pathways with mouse EECs, but only 6 with hPSC‐derived epicardial cells (Tables 1, 2). As expected, both hPSC‐derived EECs and msEECs, but not hPSC‐derived epicardial cells, were highly enriched in angiogenesis and vasculature development‐related pathways. Interestingly, the hPSC‐derived EECs and msEECs were also enriched in regulation of body fluid levels and hemostasis, perhaps resulting from their functions as a blood‐heart barrier (BHB). In addition, hierarchical clustering analysis of RNA‐seq expression data of hPSCs,^10^ hPSC‐mesoderm^10^ (Mes), hPSC‐cardiomyocytes^10^ (CMs), hPSC‐epicardial cells^10^ (Epi), hPSC‐EECs, hCMECs, and HUVECs showed that hPSC‐derived EECs were most closely related to the two endothelial cell types (hCMECs and HUVECs), and were distinct from all other cell populations as a group (Figure 5d). Next, we explored the developmental relationships between different cardiac cell types by performing principal component analysis (PCA) on the gene expression data. Undifferentiated hPSCs clustered relatively close to Mes cells, from which Epi and CMs divergently formed, in the 3D scores plot for the first 3 principal components (Figure 5e). Importantly, hPSC‐derived EECs, closer to hCMECs, were distinct from epicardial cells. Taken together, our hPSC‐derived EECs, like mouse EECs, displayed molecular signatures of endocardial endothelial cells.

**Table 1:** Gene annotations enriched in hPSC‐derived EECs and epicardial cells and murine EEC compared to undifferentiated hPSCs

GO Description	Size
Proteinaceous extracellular matrix	82
Collagen	22
Extracellular matrix	83
Organ morphogenesis	117
Positive regulation of cell differentiation	21
Extracellular matrix part	49
Skeletal development	79
Regulation of cell adhesion	28
Muscle development	88

**Table 2:** Gene annotations enriched in hPSC‐derived EECs and msEEC compared to undifferentiated hPSCs

GO Description	Size
Wound healing	40
Blood coagulation	32
Hemostasis	35
Coagulation	33
Regulation of body fluid levels	42
Bone remodeling	20
Mesoderm development	20
Extracellular matrix structural constituent	22
Regulation of angiogenesis	23
Vasculature development	47
Angiogenesis	40
Rho protein signal transduction	36
Anatomical structure formation	48
Negative regulation of cell adhesion	16
Insulin receptor signaling pathway	19
Integrin complex	17
Heart development	33
Transmembrane receptor protein serine threonine kinase signaling pathway	42
Ras protein signal transduction	64
Cell substrate adhesion	38
Small GTPase mediated signal transduction	84
Transforming growth factor beta receptor signaling pathway	32
Membrane organization and biogenesis	118
Jak stat cascade	27
Cell Matrix Adhesion	37
Small GTPase regulator activity	67
Enzyme linked receptor protein signaling pathway	122

## 3 DISCUSSION

The endocardium is the innermost endothelial layer of the heart, serving as a BHB, an endothelial layer that regulates the ionic composition of the cardiac microenvironment via passive tight junctions and active transporter systems. The endocardium also modulates myocardium performance by releasing trophic factors in response to humoral and mechanical stimuli.^26^, ^27^, ^28^ In addition to its signaling roles during heart development and regeneration, the endocardium has also been shown to contribute multi‐lineage descendants to cardiac valves, septa, hematopoiesis, and coronary blood vessels.^29^, ^30^, ^31^ Endocardial endothelium lesions and other dysfunctions are associated with various cardiovascular diseases, including parietal endocarditis, intraventricular thrombi in myocardial infarction, and hypereosinophilic endomyocardial fibrosis.^32^, ^33^ Despite the importance of the endocardium for heart development and function, the embryonic origin of endocardium and the underlying molecular mechanisms that regulate endocardium development remains poorly understood, especially in humans.

hPSCs provide a unique model to understand the developmental mechanisms regulating the specification of human cell lineages. This model enables systematic studies to identify cues that control developmental fate choices and can also produce cells for patient‐specific cell therapies. Here, we show that activation of Wnt signaling directs hPSC‐derived cardiac progenitors toward epicardial cells, some of which give rise to endothelial cells upon VEGF treatment. The resulting epicardial cell‐derived endothelial cells displayed many characteristics of endocardial endothelial cells, including expression of key EEC proteins CD31, VE‐cadherin and NFATc1, and elevated Glut‐1 expression. These hPSC‐derived EECs also express and exhibit junctional localization of barrier proteins such as occludin and ZO1, and exhibit global gene expression profiles as mouse primary EECs. Previous studies have generated epicardial cells from hPSCs and differentiated them into smooth muscle cells and cardiac fibroblasts.^11^, ^12^, ^13^ These hPSC‐derived epicardial cells had not been differentiated to endothelial cells, however. Using a WT1‐2A‐eGFP reporter cell line, we showed that WT1+ epicardial cells can give rise to endocardial‐like endothelial cells. To our knowledge, this is the first employment of an *in vitro* reporter system to confirm the epicardial‐to‐endothelial transition. Our finding is consistent with the *in vivo* observation that epicardial cells, marked by SCX/Sema3D, can give rise to NFATc1+ endocardial cells during mouse heart development.^7^

The epicardial contribution to the endocardium *in vivo* in animal models is small likely due to the fact that endocardial tubes form before the epicardium during heart development,^7^ which might also explain the low efficiency (∼2%) of epicardial‐to‐endocardial transition even with high VEGF in our hPSC model. Therefore, there may be other cardiac progenitors that contribute to the majority of the endocardium. Compared to the epicardium, mesodermal progenitor cells marked by Flk1+ are better established as origins for endocardium in both chicken and mice embryos before primary heart field formation.^34^, ^35^, ^36^, ^37^ In addition, Isl1+ second heart field progenitors were shown to give rise to the endocardium by re‐expressing Flk1.^38^, ^39^ However, generation of human endocardium from either Flk1+ or Isl1+ progenitors have not yet been described. Based on cardiac developmental studies in animal models, we expect that multiple progenitor populations may contribute to endocardial formation in humans.

A better understanding of endocardium development will assist development of novel approaches to efficiently generate functional EECs that can be used in basic and translational applications. First, EECs may provide functional improvements in engineered myocardium and heart valve tissues. Including vascular endothelial cells in human heart tissues was shown to significantly improve engraftment rates and cardiac functions in infarcted hearts.^40^, ^41^ As a BHB, EECs might provide a more unique niche to modulate cardiac contraction and support cardiac function within engineered tissues and infarcted hearts. Second, hPSC‐derived endocardium can be employed as an *in vitro* model to study molecular mechanisms that regulate endocardium development and dysfunction, as well as mechanotransduction and signaling in response to blood flow and stress. For example, Nemer and Nemer showed that inhibition of GATA5 expression in an *in vitro* model of mouse endocardial differentiation blocks endocardial cell formation.^22^ Lastly, EECs can also be used to develop *in vitro* endocardium‐myocardium co‐culture systems to study the paracrine or direct‐contact interactions as these cellular communications are crucial for normal heart development and function. Such a co‐culture system might help to identify key regulators of cardiomyocyte maturation which would improve efforts to regenerate the damaged heart. However, to make these applications practical, it will necessary to increase the purity and yield of VE‐cad+ cells from hPSC‐derived epicardial cells and to develop strategies to expand hPSC‐derived EECs. Enhancing WT1 expression levels in epicardial cells, use of a 3D culture system, addition of growth factors in addition to VEGF, and co‐culture with physiologically relevant cardiac cells may improve EEC differentiation or self‐renewal.

In summary, our results demonstrate that a subset of human endocardial endothelial cells arise from hPSC‐derived WT1+ epicardial cells *in vitro* (Supporting Information Figure S5), providing a more complete understanding of the epicardial progenitor populations that form the endothelium, and a method to produce human endocardial endothelial cells for both research and translational applications.

## 4 MATERIALS AND METHODS

### 4.1 Construction of donor plasmid and sgRNA

Dual Cas9 and sgRNA backbone was digested with BbsI restriction enzyme for rapid sgRNA cloning as previously described.^10^ Two sgRNAs targeting near the *CDH5* stop codon (1: TCAGCCAGCATCTTAAACCTGGG and 2: TTTTTGGAGGCTGTGGTGCCTGG) with a G added at the beginning were used. To generate the CDH5‐2A‐eGFP donor plasmid, DNA fragments of about 2kb in length were PCR amplified from genomic DNA before and after the stop codon of *CDH5* and were cloned into the OCT4‐2A‐eGFP donor plasmid^14^ (Addgene #31938), replacing the *OCT4* homologous arms.

### 4.2 Maintenance of hPSCs and TAT‐Cre treatment of CDH5 knock‐in hPSCs

Transgene and vector‐free hPSCs were maintained on Matrigel (Corning) or SyntheMax (BD Biosciences)‐coated plates in mTeSR1 or E8 medium (STEMCELL Technologies) according to previously published methods.^42^, ^43^ To remove the PGK‐Puro cassette from the CDH5‐2A‐eGFP cells, targeted homozygous clones were treated with 2 μM TAT Cre Recombinase (Excellgen, EG‐1001) for 6 hr in E8 medium. After 2 days, cells were singularized with Accutase (Innovative Cell Technologies) and seeded into a Matrigel‐coated 96‐well plate at a density of 100–150 cells per well. After 2 weeks, cells were subjected to PCR genotyping.

### 4.3 Electroporation

hPSCs pretreated with 10 μM ROCK inhibitor (Y27632) for 3 to 4 hr prior to electroporation. Cells were digested by Accutase at 37 °C for 8 min and 2.5–3 million singularized cells were electroporated with 3 μg gRNA1, 3 μg gRNA2, and 6 μg CDH5‐2A‐eGFP donor plasmids in 200 μl cold PBS ‐/‐ using the Gene Pulser Xcell System (Bio‐Rad) at 320 V, 200 μF, and 1,000 Ω (Time constant should be around 15 ms) in a 0.4 cm cuvette. Two electroporations were performed and in total 5–6 million cells were subsequently plated onto a Matrigel‐coated 10‐cm dish in 10 mL mTeSR1 with 10 μM Y27632. Twenty‐four hours later, and every day afterward, the medium was changed with fresh mTeSR1. Three days after electroporation, 1 μg/ml puromycin was added to the mTeSR1 for selection for about 2 weeks. Single cell clones were then picked into wells of a Matrigel‐coated 96‐well plate and subjected to PCR genotyping after 4–7 days.

### 4.4 Epicardial cell generation via modulation of canonical Wnt signaling

Day 6 cardiac progenitor cells and epicardial cells were generated as previously described.^10^, ^44^, ^45^ Briefly, cardiac progenitor cells were singularized with Accutase at 37 °C for 5 min and then seeded onto a gelatin‐coated cell culture dish at 20,000–80,000 cells/cm^2^ in LaSR basal medium (advanced DMEM/F12 with 100 µg/ml ascorbic acid) or RPMI/Vc/Ins medium (100 µg/ml ascorbic acid and 1 µg/ml human recombinant or bovine insulin) with 5 μM ROCK inhibitor Y‐27632 for 24 hr. At day 7 after initiation of differentiation, cells were treated with 3 µM CHIR99021 for 2 days in LaSR basal medium or RPMI/Vc/Ins medium. After 2 days, CHIR99021‐containing medium was aspirated and cells were cultured in LaSR basal medium or RPMI/Vc/Ins medium without CHIR99021 for 3–5 additional days.

### 4.5 Endothelial cell differentiation from hPSC‐derived epicardial cells

After maintenance in LaSR basal medium containing 0.5 µM A83‐01 for several passages, confluent hPSC‐derived epicardial cells were split 1:3 to 1:6 at a density of 0.04 to 0.08 million cells/cm^2^ using Versene (Life Technologies) or Accutase onto gelatin‐coated plates. When reaching more than 60% confluence, epicardial cells were cultured in EGM‐2 medium containing 100 ng/ml VEGF for 5 days followed by 5 days in EGM‐2 medium containing 2.5 µM A83‐01.

### 4.6 Immunostaining analysis

Cells were fixed with 4% paraformaldehyde for 15 min at room temperature and then stained with primary and secondary antibodies (Supporting Information Table S1) in PBS plus 0.4% Triton X‐100 and 5% non‐fat dry milk (Bio‐Rad). Nuclei were stained with Gold Anti‐fade Reagent with DAPI (Invitrogen). An epifluorescence microscope (Leica DM IRB) with a QImaging® Retiga 4000R camera was used for imaging. The quantification of VE‐cad+ cells in Supporting Information Figure S2b,c were performed using Image J.

### 4.7 Genomic DNA extraction and genomic PCR

QuickExtract^TM^ DNA Extraction Solution (Epicentre Cat. # QE09050) was used to rapidly extract genomic DNA from hPSCs according to the manufacturer's instructions. Genomic PCR was carried out using GoTaq Green Master Mix (Promega Cat. # M7123). PCR primer sequences are provided in the Supporting Information Table S2.

### 4.8 RNA sequencing and data analysis

Human primary cardiac microvascular endothelial cells were purchased from PromoCell. Total RNA of corresponding samples was prepared with the Direct‐zol^TM^ RNA MiniPrep Plus kit (Zymo Research) according to the manufacturer's instructions. Samples were analyzed in an Illumina HiSeq 2500 by Biotechnology Center at University of Wisconsin‐Madison. The resulting sequence reads were mapped to the human genome (hg19) using HISAT,^46^ and the RefSeq transcript levels (RPKMs) were quantified using the python script rpkmforgenes.py.^47^ Hierarchical clustering of whole transcripts was then plotted using GENE‐E. PCA was performed using PLS Toolbox 8.1 (Eigenvector Technologies). The whole transcripts were preprocessed using an auto‐scaling method (subtracting the mean from the variables and dividing by the standard deviation) to study the variance. Pathway enrichment analysis was performed using GSEA software.^48^ The gene expression data for each cell type were compared to expression data from undifferentiated hPSCs and the significantly enriched pathways (*p* < .05) were considered for further analysis. MATLAB 2013a (MathWorks Inc.) and Microsoft Excel (2013) were used to identify the unique and common pathways in different cell types. The final processed data and raw fastq files were submitted to Gene Expression Omnibus with the accession number GSE93705.

## 5 CONCLUSIONS

This study demonstrates that hPSC‐derived WT1+ epicardial progenitor cells can give rise to endothelial cells that display properties of endocardial endothelial cells. This suggests that a subset of human endocardial cells may have origins in the epicardium and identifies hPSC‐derived epicardial cells a potential source for endocardial tissue engineering and regenerative medicine.

## CONFLICT OF INTERESTS

The authors declare no competing financial interests.

## AUTHOR CONTRIBUTIONS

XB and SPP designed this study and prepared the manuscript. XB and VJB undertook experimentation and data analysis. TH, TQ, and XL contributed to study design and assisted in experiments and data analysis. All authors reviewed and approved the manuscript.

## Supporting information

Additional Supporting Information may be found online in the supporting information tab for this article.

Supporting Figures and Tables
