# Supplementary material for: Human pluripotent stem cell‐derived epicardial progenitors can differentiate to endocardial‐like endothelial cells
Source: Bioeng Transl Med. 2017 May 22;2(2):191–201. doi: 10.1002/btm2.10062 (PMC5675097; doi:10.1002/btm2.10062)
Supplement: Supplementary file 1 — Supporting Figures and Tables [file BTM2-2-191-s001.docx]

**SUPPLEMENTAL FIGURES AND LEGENDS**

**
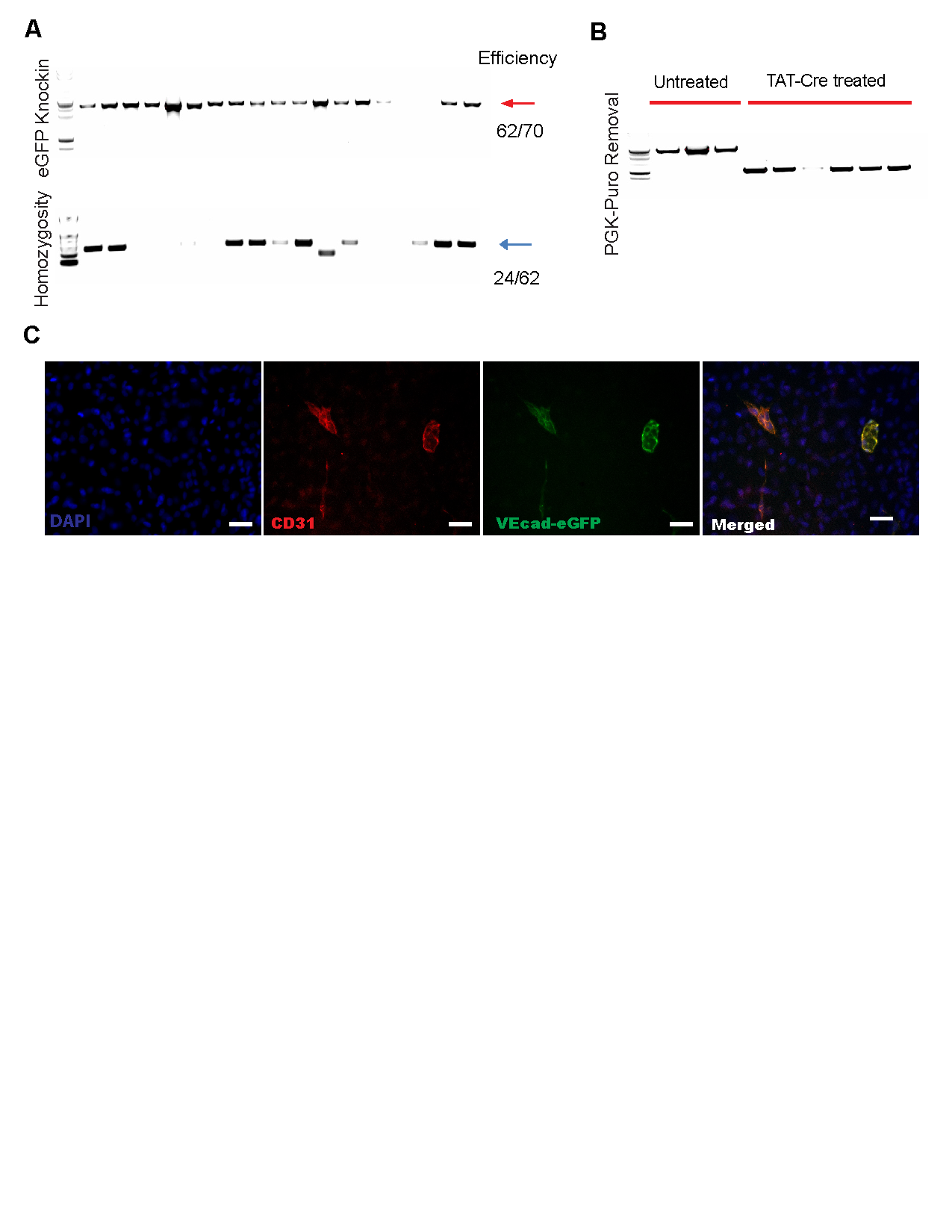
**

**Figure S1.** Generation of CDH5-2A-eGFP knockin H13 hESC lines using Cas9 nuclease. Knockin was target and as illustrated in Fig. 1A. (A) Representative PCR genotyping of hESC clones after puromycin selection. The expected PCR product for correctly targeted *CDH5* locus is ~3 kb (red arrows) and this product was observed in 62 of 70 clones analyzed. Correctly targeted clones underwent a further homozygosity assay. Clones with the PCR products of ~200 bp are heterozygous (blue arrow), and those clones without PCR products are homozygous (24/62). (B) PCR genotyping of hESC clones after TAT-Cre mediated excision of the PGK-Puro cassette. Clones with the PCR products of ~1 kb are PGK-Puro free, and those with ~3 kb contain PGK-Puro. (C) Representative CD31 and eGFP dual immunostaining images of CDH5-2A-eGFP hPSC-derived endothelial cells after excision of the PGK-Puro cassette. Scale bars, 50 µm.

**
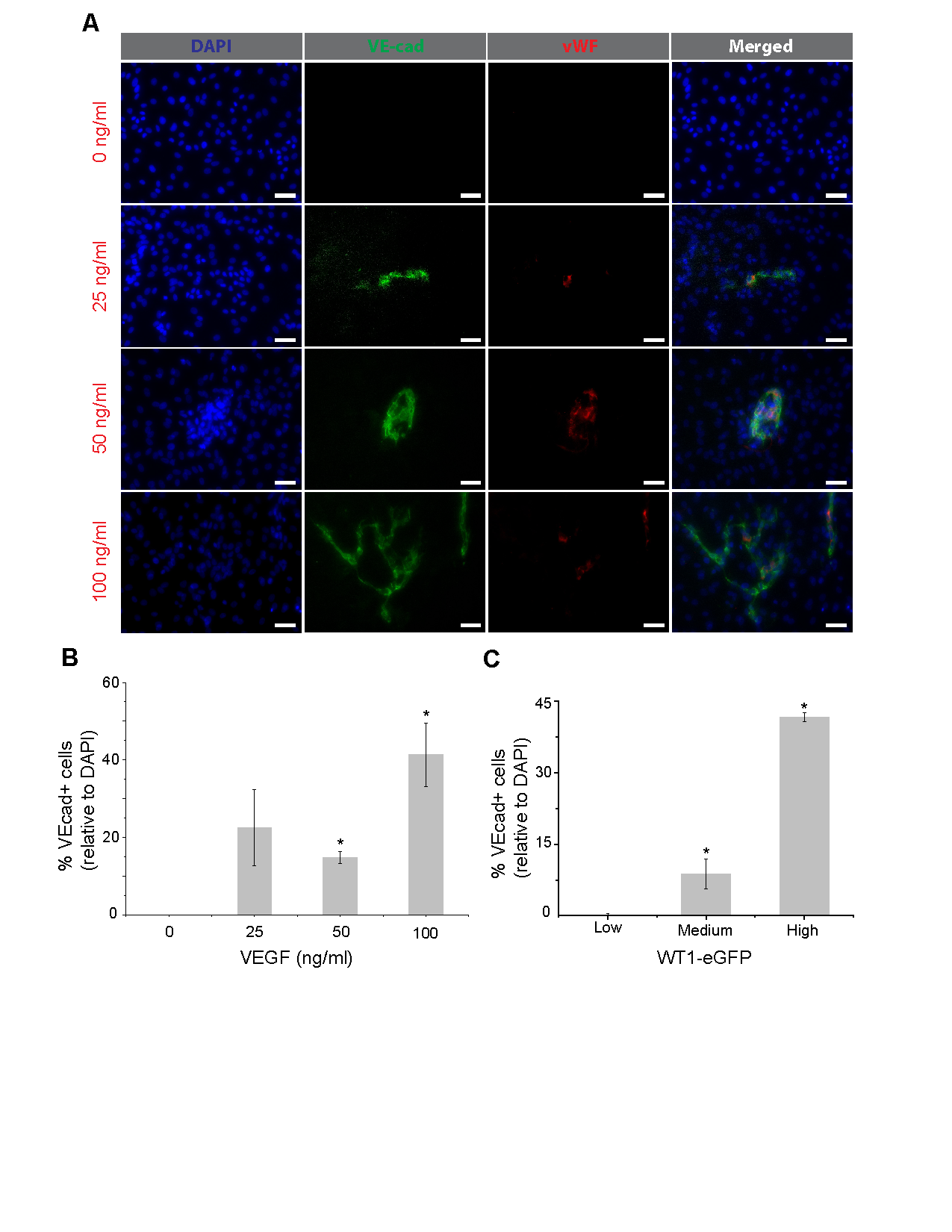
**

**Figure S2.**VEGF treatment enhanced the generation of endothelium from WT1+ epicardium. Epicardial cells were treated as illustrated in Figure 2C with VEGF at the indicated concentrations. Representative immunostaining images and quantitative analysis data are shown in A and B, respectively. Scale bars, 50 µm. Data are represented as mean ± s.e.m of three independent replicates. *P < 0.05, indicated treatment versus 0 ng/ml VEGF. (C) Quantitative analysis data of VE-cadherin+ cells from epicardial cells with low, medium, or high WT1-eGFP expression (Figure 2E) is shown. Data are represented as mean ± s.e.m of three independent replicates. *P < 0.05, indicated treatment versus low WT1-eGFP.

**
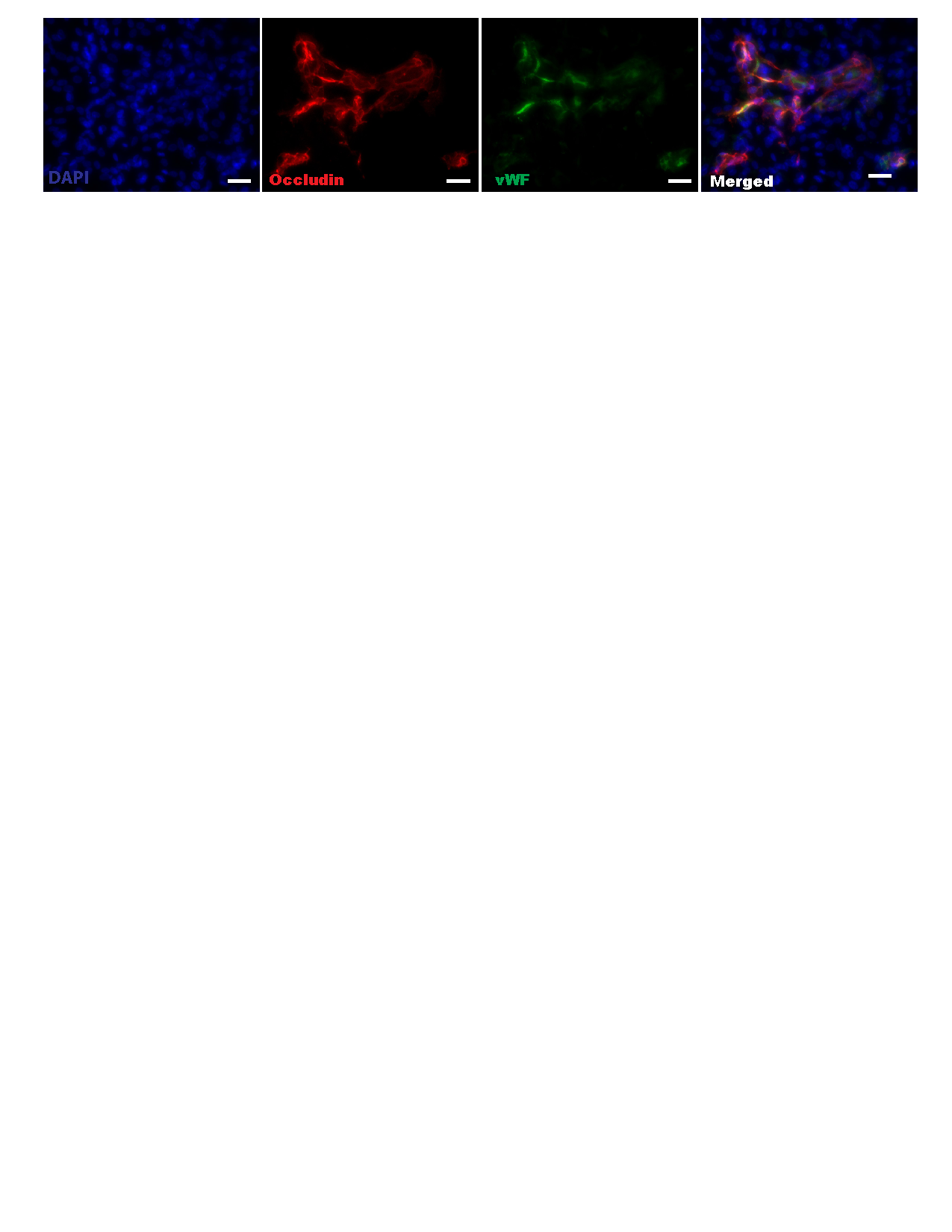
**

**Figure S3.** Representative occludin and vWF dual immunostaining images of WT1-2A-eGFP ES03-derived endocardial endothelial cells. Scale bar, 50 µm.

**
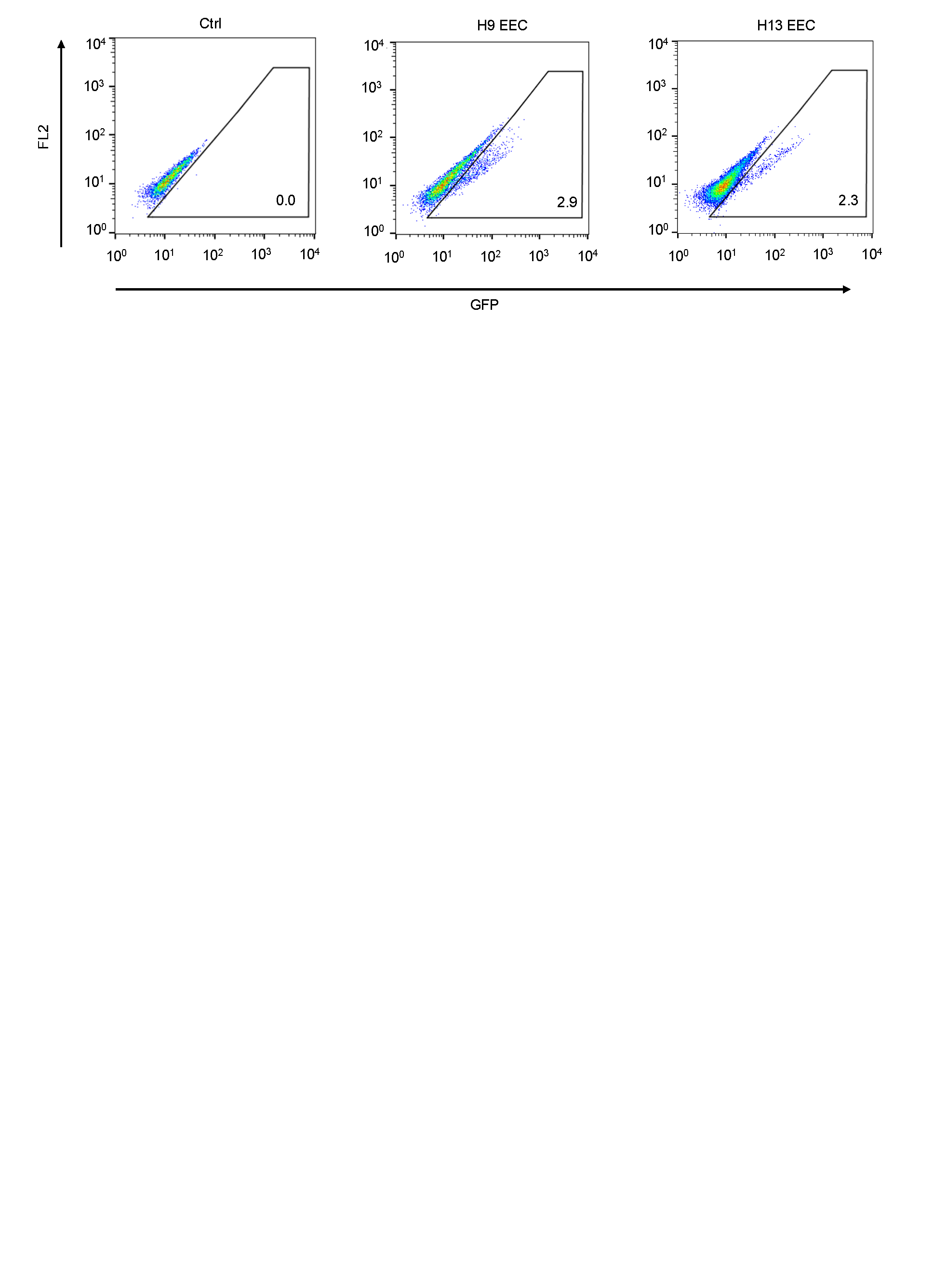
**

**Figure S4.** Representative eGFP expression in day 10 H9 and H13 VE-cad-2A-eGFP hPSC-derived cultures differentiated as shown in Figure 3A. Numbers indicate the percentage of cells in the gated regions.

**
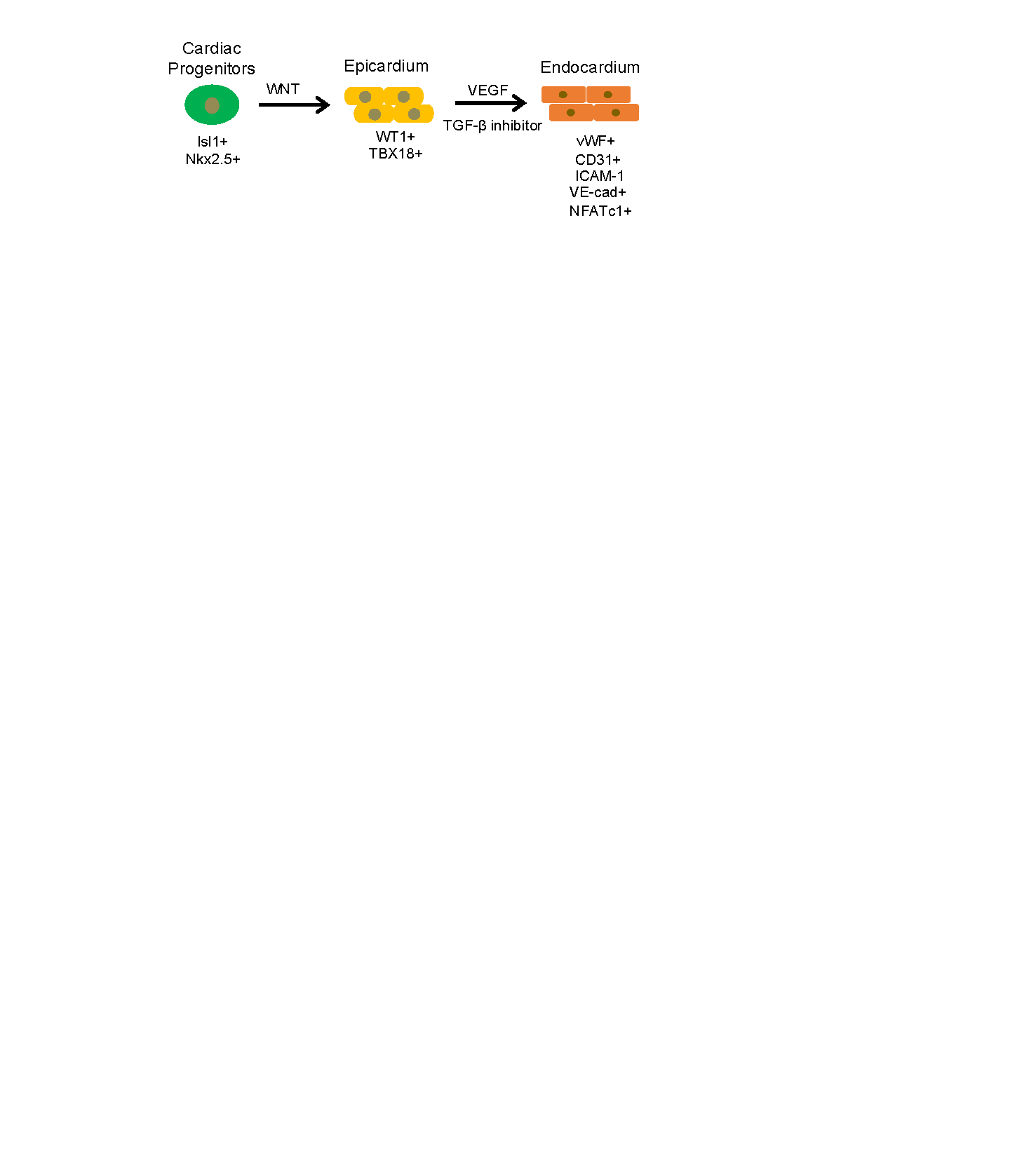
**

**Figure S5.** Schematic model highlighting the specification of endocardial-like endothelial cells from hPSC-derived epicardial cells and associated markers of differentiation state.

**SUPPLEMENTAL TABLES**

**Table S1. Antibodies used in this study**

| **Antibody** | **Source/Isotype/clone /cat. no.** | **Concentration** |
| --- | --- | --- |
| vWF | Dako/Rabbit IgG/A008202-5 | 1:500 |
| ICAM-1 | DSHB/Mouse IgG1/P2A4 | 1:30 |
| CD31-APC | Miltenyi Biotec/Mouse IgG1/130-092-652 | 1:250 |
| NFATc1 | ThermoFisher/Mouse IgG1/MA3-024 | 1:100 |
| Glut-1 | ThermoFisher/Mouse IgG2a/ms-10637 | 1:100 |
| Occludin | Invitrogen/Mouse IgG1/33-1500 | 1:50 |
| WT1 | Abcam/Rabbit IgG/ab89901 | 1:250 |
| TBX18 | Sigma-Aldrich/Rabbit IgG/HPA029014 | 1:200 |
| VE-cadherin | Santa Cruz/Mouse IgG1/F-8/sc9989 | 1:100 |
| CD31 | ThermoFisher/Rabbit IgG/RB-10333-P | 1:100 |
| ALDH1A2 | Sigma-Aldrich/Rabbit IgG/HPA010022 | 1:50 |
| GFP | DSHB/Mouse IgG1/12E6 | 1:20 |
| Secondary Antibody | Alexa 488 Chicken anti-Gt IgG/A-21467 | 1:1,000 |
| Secondary Antibody | Alexa 488 Chicken anti-Rb IgG/A-21441 | 1:1,000 |
| Secondary Antibody | Alexa 488 Goat anti-Ms IgG1/A-21121 | 1:1,000 |
| Secondary Antibody | Alexa 488 Goat anti-Rb IgG/A-11008 | 1:1,000 |
| Secondary Antibody | Alexa 594 Goat anti-Ms IgG2b/A-21145 | 1:1,000 |
| Secondary Antibody | Alexa 594 Goat anti-Rb IgG/A-11012 | 1:1,000 |
| Secondary Antibody | Alexa 647 Goat anti-Ms IgG2b/A-21242 | 1:1,000 |
| Secondary Antibody | Alexa 647 Goat anti-Rb IgG/A-21244 | 1:1,000 |

Table S2. Oligonucleotide primers used in this study

| **Genes** | **Sequences (5' - 3')** | **Size (bp)/Tm (°C)** |
| --- | --- | --- |
| *CDH5 KI (Red)* | **F:**GGCCTTGGTGTTTCCAGATA  **R:**TGGTGCAGATGAACTTCAGG | 2597/60 |
| *CDH5 KI (Blue)* | **F:**TCCGACTCTGACGTGGATTA  **R:**AAGGCTGATGAGGTCTCTGG | 250/60 |
